# Cistanoside F Ameliorates Lipid Accumulation and Enhances Myogenic Differentiation via AMPK-Dependent Signaling in C2C12 Myotubes

**DOI:** 10.3390/cells14120874

**Published:** 2025-06-10

**Authors:** Meng-Ling Ma, Ze-Ling Tang, Li-Ping Chen, Xiang-Nan Qin, Ke-Fei Xiao, Wei-Liang Zhu, Yong Zhang, Zhang-Bin Gong

**Affiliations:** 1 Department of Biochemistry, School of Integrative Medicine, Shanghai University of Traditional Chinese Medicine, Shanghai 201203, China; 12024011@shutcm.edu.cn (M.-L.M.);; 2 Shanghai Institute of Materia Medica, Chinese Academy of Sciences, Shanghai 201203, China

**Keywords:** cistanoside F, sarcopenic obesity, AMPK pathway, adipogenesis, C2C12 cells

## Abstract

Sarcopenic obesity (SO) is a metabolic disorder for which no effective pharmacological treatments are currently available. Cistanoside F (Cis), a phenoxyethanol-derived compound, remains relatively unexplored in the context of lipid metabolism regulation, as well as its potential mechanisms and therapeutic applications in metabolic disorders. Consequently, this study aimed to evaluate the potential of Cis in ameliorating the pathological manifestations of SO in C2C12 cells. Two classical adipogenic differentiation models using C2C12 cells were employed to quantitatively assess the ability of Cis to inhibit lipid droplet formation, utilizing Oil Red O staining coupled with high-content imaging analysis. Markers associated with adipogenic and myogenic differentiation were examined using quantitative real-time PCR and Western blotting. Our experimental findings demonstrated that Cis significantly attenuated lipid droplet accumulation and promoted muscle protein synthesis via the modulation of PPARγ, ATGL, CPT1b, and UCP1 expression during lipogenic differentiation of C2C12 cells. Cis significantly upregulated the phosphorylation and expression levels of key metabolic regulators, including p-AMPK/AMPK, p-ACC1/ACC1, and MHC. We identified a positive regulatory feedback mechanism between AMPK signaling and MHC expression in the adipogenic differentiation model, suggesting that Cis exerts its therapeutic effects through AMPK-dependent pathways. This is the first study to provide the first experimental evidence supporting the therapeutic potential of Cis for metabolic regulation, targeting adiposity reduction and muscle mass enhancement. Furthermore, Cis exhibited potent anti-inflammatory properties, as demonstrated by its ability to significantly downregulate proinflammatory mediators, including IL-6 and p-NF-κB/NF-κB, during adipogenic differentiation. These novel findings regarding the anti-inflammatory mechanisms of Cis will form the basis for our subsequent in-depth mechanistic investigations.

## 1. Introduction

Individuals with obesity demonstrate considerable impairments in skeletal muscle quality and function, primarily due to the progressive deterioration of muscle architecture and lipid infiltration [1,2]. According to the European Society for Clinical Nutrition and Metabolism and the European Association for the Study of Obesity, sarcopenic obesity (SO) is defined as the concurrent manifestation of excessive adiposity and sarcopenia [3], the prevalence of which is increasing rapidly. The pathophysiology of SO involves complex biological processes characterized by increased whole-body adiposity and ectopic fat deposition within skeletal muscle tissues, particularly intramuscular fat (IMF). IMF accumulation occurs through both intramyocellular and extramyocellular mechanisms, encompassing lipid droplet formation in myoblasts and adipocytes, ultimately leading to reduced muscle mass and compromised muscle quality [4]. In contrast to isolated obesity or sarcopenia, SO is a unique clinical entity associated with multiple comorbidities, including accelerated sarcopenia progression and physical disability [5]. SO exerts more severe metabolic consequences and higher mortality risks than either isolated obesity or sarcopenia alone. Therapeutic interventions for weight reduction, including dietary modifications [6] and bariatric surgery [7], are associated with major challenges in preserving lean body mass, potentially exacerbating sarcopenia conditions. However, although the worldwide prevalence of obesity has increased substantially [8,9], the precise mechanisms underlying obesity-mediated sarcopenia progression remain poorly understood.

The therapeutic paradigm for SO involves a dual approach, targeting both adiposity reduction and muscle mass preservation. Current pharmacological interventions approved by the US Food and Drug Administration, including liraglutide and semaglutide, demonstrate limited efficacy in addressing the complex pathophysiology of SO. Although liraglutide has considerable lipid-lowering properties, its clinical application does not substantially impact lean body mass preservation. Moreover, semaglutide, a double-acting agent for lipid-lowering and muscle mass maintenance, is associated with significant adverse effects, including hypoglycemia, gastrointestinal complications, pancreatitis, and diabetic retinopathy in clinical settings [10,11]. These limitations underscore the need to develop safer therapeutic agents that simultaneously address adiposity reduction and muscle mass enhancement. Phenylethanoid glycosides (PeGs) represent a promising class of bioactive compounds characterized by a β-glucose core structure with glycosidic linkages to phenylacrylates and α-hydroxyphenethyl moieties. These naturally occurring secondary metabolites, frequently modified with acetyl, caffeoyl, and various sugar groups, are ubiquitously distributed across >200 plant species spanning 23 families—including numerous pharmacologically important medicinal plants [12,13,14]. Extensive pharmacological studies have revealed that PeGs possess a broad spectrum of biological activities, including analgesic, sedative, anti-inflammatory, antioxidant, anticancer, immunomodulatory, and neuroprotective properties, along with cardiovascular and cerebrovascular regulatory properties.

Cistanoside F (Cis), a prominent bioactive constituent in PeGs, degrades into caffeic acid, hydroxyl alcohol, and other phenolic acid derivatives via metabolic reactions, such as methylation, acetylation, hydroxylation, and hydrolysis in vivo. Cistanosides are caffeic acid derivatives, representing a class of phytochemicals ubiquitously distributed in various medicinal plants. They predominantly exist as carbohydrate esters and have diverse pharmacological profiles, including immunomodulatory, choleretic, and antimicrobial properties. Notably, comparative studies have demonstrated that these derivatives exhibit superior free radical scavenging capacity compared with α-tocopherol—as evidenced by their enhanced activity against 1,1-diphenyl-2-picrylhydrazyl radicals and superoxide anions generated via the xanthine/xanthine oxidase system [15]. Cis’s molecular structure is characterized by the presence of both ester and glycosidic bonds, linking a glycosyl unit, rhamnosyl moiety, and caffeoyl group. This unique structural configuration has been identified in multiple plant species across various genera, including *Cistanche*, *Orobanche*, *Scrophularia*, and *Callicarpa* [16,17,18,19,20]. Previous pharmacological studies have established the antioxidant properties of Cis and its hepatoprotective effects against liver injury [21]. However, the potential therapeutic applications of Cis in modulating lipid metabolism and promoting myogenesis remain unexplored, representing a major gap in current knowledge.

Sarcopenia is a muscular disorder characterized by progressive and extensive loss of skeletal muscle mass and strength [22]. It has been reported that SO is closely related to changes in muscle composition, which is not limited to the loss of body weight and muscle mass, e.g., fat infiltration into the muscles affects myogenesis and leads to a decrease in the quality and working capacity of the muscles [23]. Myogenic differentiation and the formation of myotubes play important roles in myogenesis [23,24,25]. However, the function of skeletal myogenesis and its maintenance warrants further investigation. Reduction in muscle fiber quantity is the primary contributor to sarcopenia [26]. Subsequent differentiation of myoblasts—characterized by the expression of markers such as myosin heavy chain (MHC), troponin, and myogenin—eventually halts new myoblast proliferation. This process culminates in the fusion of these differentiated cells with existing myofibers, resulting in new myofiber formation [27,28]. Peroxisome proliferator-activated receptor gamma (PPARγ) is a key marker of adipogenic differentiation [29]. The transcriptional remodeling of mature adipocytes is closely related to uncoupling protein 1 (UCP1) and adipose triglyceride lipase (ATGL) [30,31]. AMP-activated protein kinase (AMPK) signaling is a crucial regulator of lipid metabolism. AMPK modulates the activities of downstream acetyl-CoA carboxylase (ACC) and carnitine palmitoyltransferase 1 (CPT1), leading to various biological consequences. ACC is a crucial enzyme in fatty acid biosynthesis, particularly in the catalysis of the acetyl-CoA-to-malonyl-CoA conversion, which is a major part of new fat formation [32]. ACC can be phosphorylated under AMPK catalysis, deactivating and inhibiting malonyl CoA and new fat production and promoting the oxidative metabolism of fatty acids to further regulate lipid homeostasis [33,34,35].

Obesity is strongly linked to an increase in chronic systemic inflammation, both of which accelerate sarcopenia risk and worsen systemic disease prognosis [36]. A detrimental cycle involving the persistence of elevated adipose tissue and inflammation in skeletal muscle may contribute to SO onset and progression. The infiltration of fat cells, also known as lipotoxicity, can promote toxic adipocytokine release to affect the function of muscle cells. Adipocytokines, including well-characterized mediators such as nuclear factor kappa-B (NF-κB) and interleukin (IL) 6, are strongly implicated in sarcopenia pathogenesis. In the context of obesity, adipose tissue undergoes significant structural and functional alterations, including adipocyte hypertrophy, hyperplasia, and activation. These changes lead to the accumulation of immune cells, including proinflammatory macrophages, as well as the dysregulated secretion of various adipokines. These mechanisms collectively create a proinflammatory microenvironment, driven by a complex interplay of molecular and cellular pathways [37,38]. The intricate crosstalk among muscle tissue, adipose tissue, and various external factors likely contributes to a cascade of events leading to detrimental long-term health risks.

Here, we explored the role of Cis in promoting myogenic differentiation and reducing lipid accumulation. Our findings demonstrated that Cis could considerably activate AMPK, as well as directly modulate ACC activity and upregulate MHC expression. Furthermore, Cis was noted to play a pivotal role in enhancing myogenic factor expression, suppressing lipid synthesis, and promoting fat mobilization. In summary, we elucidated the regulatory mechanisms of Cis in myogenesis and adipogenesis, providing novel insights into muscle wasting pathogenesis in individuals with obesity. Our results highlight Cis as a potential therapeutic target for addressing muscle wasting and SO.

## 2. Materials and Methods

### 2.1. Reagents and Antibodies

An oil red staining kit, cell counting kit 8 (CCK8), RIPA lysis buffer, BCA protein concentration assay kit, ROS assay kit, and MMP assay kit were purchased from Beyotime (Shanghai, China; #C0037; #P0039; #P0009; #S0033S; #C2001S; #C2006). Triglyceride (TG) test, total cholesterol (TCHO) test, low-density lipoprotein (LDL) assay, and high-density lipoprotein cholesterol (HDL) assay kits were procured from Jiancheng Biotech (Nanjing, China; #A1110; #A1111; A1113; #A1112). Dulbecco’s modified Eagle’s medium (DMEM) and fetal bovine serum (FBS) were purchased from Thermo Fisher Scientific (San Diego, CA, USA; #11995; #10099). Antibodies against p-ACC1 (Ser79), ACC1, p-AMPK (Thr172), AMPK, ATGL, and MHC were purchased from Abcam (Cambridge, UK; #ab68191; #ab45174; #ab133448; #ab207442; #ab240381; #ab37484; 1:1000). Antibodies targeting β-actin, PPARγ, p-NF-κB (Ser536), and NF-κB were obtained from Cell Signaling Technology (Beverly, MA, USA; #8457; #2435; #3033; #8242; 1:1000). Antibodies against UCP1, CPT1b, ACC2, Desmin, and PGC-1α were purchased from Boster Biotech (Wuhan, China; #M00255; #A03558; #A03668; #PB9105; #BM4898; 1:1000). Cistanoside F, AICAR, Compound C, LPS, isobutylmethylxanthine, dexamethasone, rosiglitazone, insulin, and palmitic acid were obtained from MCE (Princeton, NJ, USA; #HY-N4220; #HY-13417; #HY-13418A; #HY-D1056; #HY-12318; #HY-14648; #HY-17386; #HY-P0035; #HY-N2341) and all primers were procured from Sangon (Shanghai, China).

### 2.2. Cell Culture and Treatments

C2C12 cells (American Type Culture Collection, Rockville, MD, USA) were plated on a 100-mm culture dish at (3–4) × 10^6^ per 20 mL of medium and cultured in M1 medium (i.e., DMEM containing 100 U/mL penicillin, 100 µg/mL streptomycin, and 10% FBS) at 37 °C in a 5% CO_2_ incubator. Cells were regularly subcultured before reaching 70% confluency, and the passage number was <8. The vehicle group of each model was cultured in M1, and an equal volume of DMSO solution medium was added for the same period of time to ensure the stability of the model.

To induce cell adipogenic differentiation, we used the IB+R+IN+D and PA models. For the IB+R+IN+D model, the medium was gradually replaced with stage I differentiation medium of M1 medium supplemented with 0.5 mM isobutylmethylxanthine (IB), 150 µM dexamethasone (D), 85 nM insulin (IN), and 30 µM rosiglitazone (R) for 2 days, followed by 85 nM IN and 30 µM R for an additional 6 days [39,40,41]. For the PA model, C2C12 cells were induced with M1 after reaching the contact inhibition stage. Then, the cells were incubated with 2%HOS for 3 days and DMEM containing 1% FBS and 100 mM BSA-conjugated palmitic acid (PA) for 48 h until the induction of lipid deposition in myotubes.

To induce cell inflammatory differentiation, we used the LPS model. Cells were treated with M1 until 70% confluency. Next, they were incubated with 2% horse serum for 3 days until muscle duct differentiation and then with 200 ng/mL lipopolysaccharide (LPS) for 24 h.

**Figure 1:**
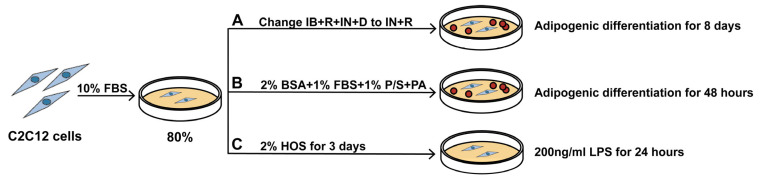
Cis treatment protocol. C2C12 cells were cultured in 10% FBS medium at a density of 80% (**A**) 0.5 mM isobutylmethylxanthine, 150 µM dexamethasone, 85 nM insulin, and 30 µM rosiglitazone (IB+R+IN+D) were used for 2 days, followed by 85 nM insulin and 30 µM rosiglitazone (IN+D) for 6 days. (**B**) Palmitic acid (PA) was used with 2% BSA, 1% FBS, and 1% penicillin/streptomycin for 48 h. (**C**) Lipopolysaccharide (LPS) was used for 24 h after 2% HOS for 3 days in C2C12 cells.

To observe the effect of Cis, all cell models were treated with 1 or 10 µM Cis over different durations (Figure 1).

### 2.3. Cell Viability Assay

C2C12 cell viability was assessed using the CCK8 assay. In brief, the cells were treated with various concentrations (0, 1, or 10 µM) of Cis for 24, 48, or 72 H, followed by treatment with the CCK8 solution. Finally, the optical density of the resulting color was measured at 450 nm under a microplate reader.

### 2.4. Oil Red O Staining and High-Content Analysis

C2C12 cells were washed with PBS, fixed with 4% paraformaldehyde for 10 min, and covered with staining washing solution for 20 s. Next, the cells were covered with the oil red O staining working solution for 30 min. Finally, the cells were removed, washed, observed, and photographed under a microscope, and High-Content Analysis was used to measure them.

### 2.5. Lipid Tests

TG, TCHO, LDL, and HDL were determined using the relevant kits according to the manufacturer’s instructions.

### 2.6. Reverse Transcription Quantitative Polymerase Chain Reaction

The total RNA was extracted using RNAiso Plus and used to synthesize cDNA. This cDNA was used to perform quantitative polymerase chain reaction (PCR) using a SYBR Premix Ex Taq kit. We used the following PCR system: 2× SYBR Premix Ex Taq II (10 μL), forward primer (0.4 μL), reverse primer (0.4 μL), TB Green Premix Ex Taq II (5 μL), and DEPC-ddH_2_O (to make up the total volume 10 μL). The following amplification conditions were used: 45 cycles of 95 °C for 30 s, 95 °C for 5 s, 61 °C for 31 s, followed by 95 °C for 15 s, 60 °C for 60 s, and 95 °C for 15 s. We used the 2^−ΔΔCt^ method to calculate the change in mRNA expression; ΔΔCt was calculated as follows: ΔCt_Sample − ΔCt_Negative Control (Table 1).

### 2.7. Western Blotting

C2C12 cell protein was extracted using cold RIPA buffer containing protease inhibitor (100×) and phosphatase inhibitor (Sangon Biotech, Shanghai, China). The total protein concentration was determined using a BCA protein concentration assay kit. Next, 20–40 µg of protein was separated through sodium dodecyl sulfate polyacrylamide gel electrophoresis (Bio-Rad, Hercules, CA, USA) and transferred onto nitrocellulose membranes. The membranes were blocked with 5% non-fat dry milk in Tris-buffered saline with Tween 20 at 37 °C for 90 min. Next, the membranes were incubated with primary antibodies at 4 °C overnight, followed by incubation with the secondary antibody at room temperature for 90 min. Finally, the membranes were exposed to ECL Western Blotting Substrate reagent (Thermo, Waltham, MA, USA).

### 2.8. ROS and Mitochondrial Membrane Potential Detection (MMP)

According to the instructions, ROS, TMRE, and JC-1 were added to the 24-well plate, and fluorescence photography was conducted after incubation in a 37 °C incubator for 30 min. For the detection, the FITC channel was selected for ROS and JC-1 monomer, and the Cy3 channel was selected for the TMRE and JC-1 aggregate. MMP was evaluated by TMRE and JC-1 fluorescence intensity. Fluorescence spectroscopy was used for mapping.

### 2.9. Immunofluorescence

C2C12 cells were cultured in 24-well plates with a round coverslip and fixed in 4% paraformaldehyde at room temperature and blocked with 5% normal goat serum and 0.3% TritonX-100 (Sigma-Aldrich, St. Louis, MO, USA) at room temperature for 1 h. After blocking, cells were incubated with a primary antibody at 4 °C overnight. After washing with PBS three times, cells were incubated with secondary Goat Anti-Rabbit IgG (H+L) Antibody (Alexa Fluor^®^ 568 Conjugate (Invitrogen, Carlsbad, CA, USA)) for 1 h in the dark. Nuclei were stained with DAPI. Fluorescence microscopy (Olympus, Tokyo, Japan) was used for analysis.

### 2.10. Enzyme-Linked Immunosorbent Assay (ELISA)

Cell culture supernatants were collected from differentiated and cultured cells, and the interleukin-6 (IL-6) level was measured using enzyme-linked immunosorbent assay (ELISA) kits according to MultiSciences Biotech’s instructions (Hangzhou, China).

### 2.11. Molecular Docking

The Cis structure file was downloaded from PubChem as a ligand, and the files of the related proteins, as receptors, were downloaded from UniProt. All files were converted to the pdbqt mode by using the RCSB Protein Data Bank and Autodock (AMPKα PDB ID: 6c9h). PyMOL 2.6 (Schrödinger, Inc., New York, NY, USA) was used as drawing software to visualize structure and docking.

### 2.12. Statistical Analysis

All experiments were conducted three or six times to ensure reliable results. All data, presented as means ± standard deviations (SDs), were analyzed using SPSS (version 21.0). Data were plotted using GraphPad Prism 9. One-way analysis of variance was performed after confirming the normal distribution and homogeneity of variance among groups. *p* < 0.05 was considered to indicate statistical significance.

## 3. Results

### 3.1. Cis Enhances C2C12 Myogenic Differentiation in Two Adipogenic Differentiation Models

**Figure 2:**
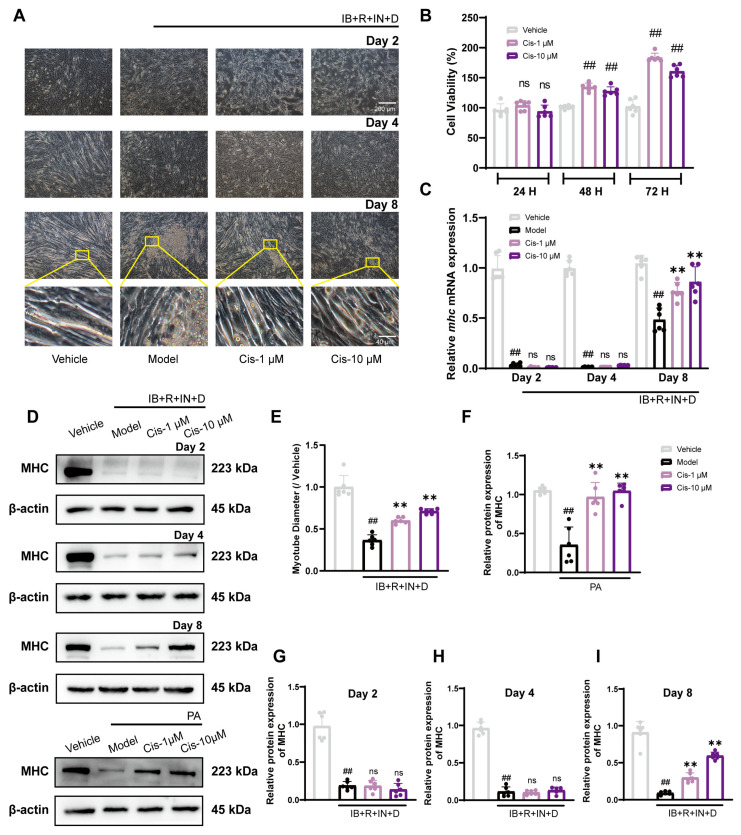
Muscle cell content and marker expression after Cis administration. (**A**,**E**) Muscle tube diameter was observed through bright-field microscopy and measured. (**B**) Cell viability was detected using the CCK8 assay for 24, 48, and 72 H. (**C**) qRT-PCR for *mhc* mRNA on days 2, 4, and 8. (**D**,**F**–**I**) MHC expression in the IB+R+IN+D model on days 2, 4, and 8 and in the PA model after 48 H. n = 6. ^##^ *p* < 0.01, compared with the vehicle group; ** *p* < 0.01, compared with the model group. ns, no significance.

To assess the safety profile of Cis, we used the CCK8 assay. The results demonstrated that it could enhance the C2C12 cell viability in a time-dependent manner, with particularly notable effects observed at 48 and 72 H (Figure 2B). In the IB+R+IN+D model, myotube diameter recovered significantly (Figure 2A,E), and Cis upregulated *Mhc* mRNA expression on day 8 (Figure 2C) but not on days 2 and 4. In the PA model, Cis effectively increased MHC expression at 48 H (Figure 2D,F–I). To sum up, Cis dose-dependently increased C2C12 cell diameter and viability.

### 3.2. Cis Reduces Intracellular Lipid Levels in C2C12 Cells

**Figure 3:**
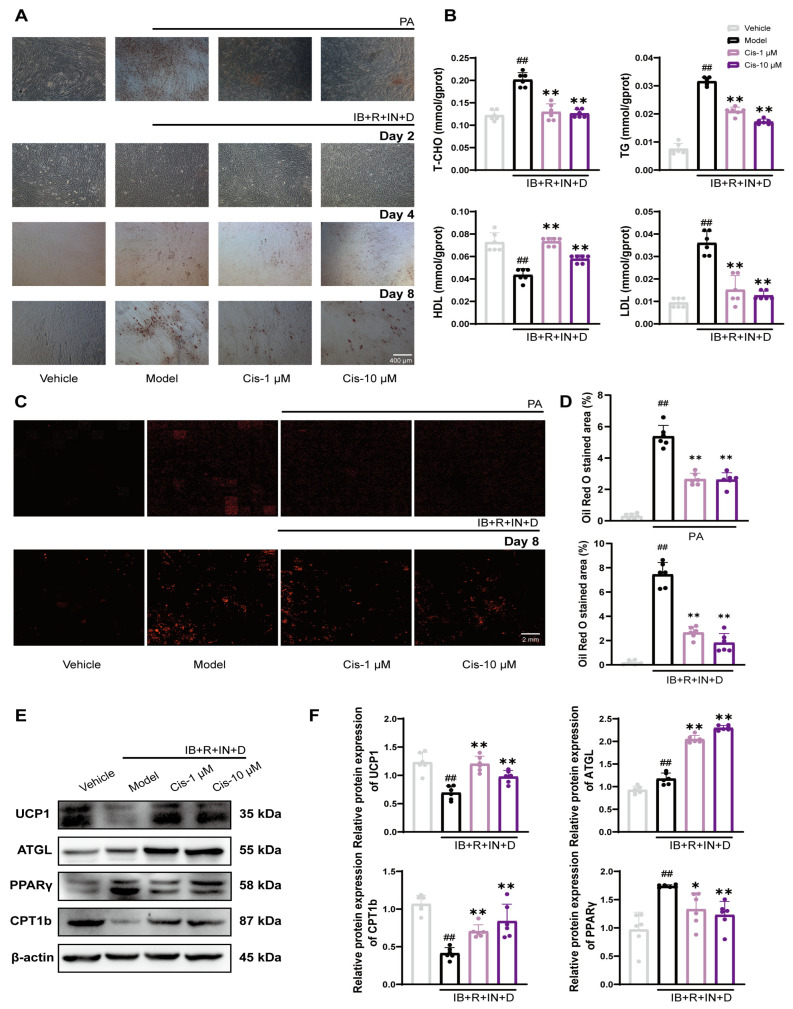
Effects of Cis on lipid content in C2C12 cells. (**A**) Lipid droplet content of two lipid differentiation models of C2C12 cells, measured using oil red staining and light microscopy. (**B**) C2C12 intracellular lipid drop content detected using TG, TCHO, LDL, and HDL detection kits. (**C**,**D**) Lipid droplets in two models of significant time of lipid differentiation were measured by a high-content instrument. (**E**,**F**) CPT1b, PPARγ, ATGL, and UCP1 expression in the IB+R+IN+D model on day 8. n = 6. ^##^ *p* < 0.01, compared with the vehicle group; * *p* < 0.05, ** *p* < 0.01, compared with the model group. ns, no significance.

To investigate the potential inhibitory effect of Cis on adipogenic differentiation in C2C12 cells, we performed oil red O staining in two experimental models. Light microscopy revealed that Cis effectively reduced lipid droplet aggregation (Figure 3A), particularly in the IB+R+IN+D model on day 8 and the PA model after 48 H. Consequently, we selected the day-8 IB+R+IN+D model as the focus for subsequent experiments. Consequently, day 8 of the IB+R+IN+D model was selected as the primary focus for subsequent experiments. To ensure objectivity and minimize human bias, we employed high-content imaging analysis to quantify the intracellular lipid droplet content, with each image reconstructed from ≥100 acquisition angles. The results consistently demonstrated that Cis effectively decreased the intracellular lipid droplet content (Figure 3C,D). TG, TCHO, LDL, and HDL detection assays confirmed that Cis significantly modulated lipid metabolism (Figure 3B). To validate the lipid-lowering efficacy of Cis further, the expression of the key lipid metabolism markers UCP1, ATGL, PPARγ, and CPT1b and the results demonstrated that Cis effectively ameliorated cell lipid deposition (Figure 3E,F). Taken together, these findings demonstrated that Cis significantly reduced intracellular lipid accumulation; the day-8 IB+R+IN+D model is the key focus of our following research.

### 3.3. Cis Effectively Reduces ROS Level and Protects Mitochondrial Function

**Figure 4:**
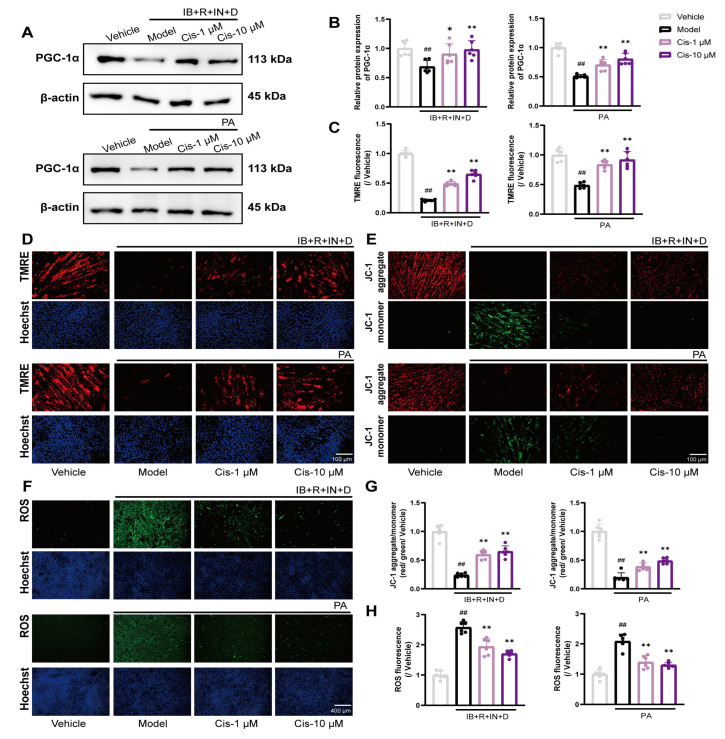
Expression of PGC-1α, MMP, and ROS in two adipogenic models after Cis administration. (**A**,**B**) PGC-1α expression. (**C**,**D**) Immunofluorescence of TMRE, the statistic was evaluated by fluorescence spectroscopy, at λ_excitation_ = 550 nm and λ_emission_ = 575 nm. (**E**,**G**) Immunofluorescence of JC-1, statistic was evaluated by fluorescence spectroscopy, JC-1 monomer at λ_excitation_ = 490 nm and λ_emission_ = 530 nm and JC-1 aggregate at λ_excitation_ = 525 nm and λ_emission_ = 590 nm. (**F**,**H**) Immunofluorescence of ROS, the statistic was evaluated by fluorescence spectroscopy, at λ_excitation_ = 488 nm and λ_emission_ = 525 nm. n = 6. ^##^ *p* < 0.01, compared with the vehicle group; * *p* < 0.05, ** *p* < 0.01, compared with the model group.

To verify the effect of Cis on mitochondrial function, ROS and MMP levels were evaluated using fluorescence microscopy and fluorescence spectroscopy in two cell adipogenic differentiation models. The results showed that Cis treatment effectively inhibits the ROS overproduction and MMP decrease (Figure 4C–H). As a key marker of mitochondrial function, Cis significantly upregulated the expression of PGC-1α (Figure 4A,B). Our data indicated that Cis effectively reduces ROS levels and protects mitochondrial function.

### 3.4. Role of the Classical AMPK/ACC1 Pathway in Lipid Differentiation

**Figure 5:**
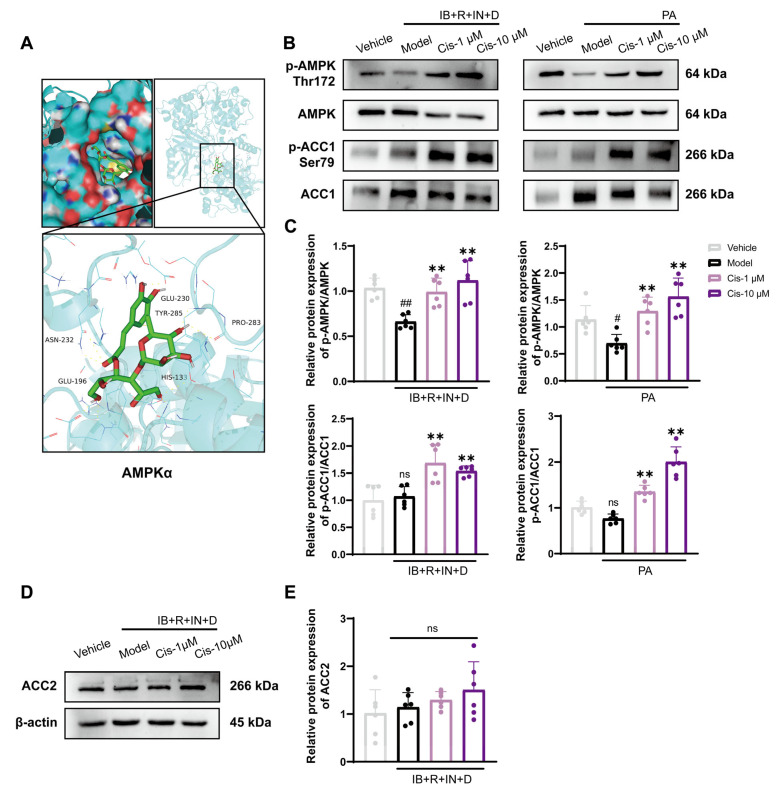
Expression of p-AMPK/AMPK and its downstream proteins p-ACC1/ACC1 after Cis administration. (**A**) Molecular docking for the AMPKα–Cis correlation. (**B**,**C**) p-AMPK/AMPK and p-ACC1/ACC1 expression in two lipogenic differentiation models. (**D**,**E**) ACC2 expression in the IB+R+IN+D model. n = 6. ^#^ *p* < 0.05, ^##^ *p* < 0.01, compared with the vehicle group; ** *p* < 0.01, compared with the model group. ns, no significance.

To further elucidate the mechanism through which Cis reduces lipid deposition, we focused on AMPK, a central regulator of lipid metabolism. Molecular docking analysis revealed that Cis binds stably within the active pocket of AMPK, with a binding energy of −8 kcal/mol, suggesting a strong compound–target protein interaction (Figure 5A). As such, we used two cell adipogenic differentiation models and found that 1 and 10 μM Cis effectively upregulated the expression of p-AMPK/AMPK and its downstream proteins p-ACC1/ACC1 (Figure 5B,C). Analysis of the other ACC isoform revealed that Cis specifically had no effect on ACC2 expression (Figure 5D,E). Taken together, these results revealed that Cis mitigates abnormal lipid deposition by activating the AMPK pathway.

### 3.5. Cis Effectively Improves the Expression of MHC Associated with AMPK Pathway

**Figure 6:**
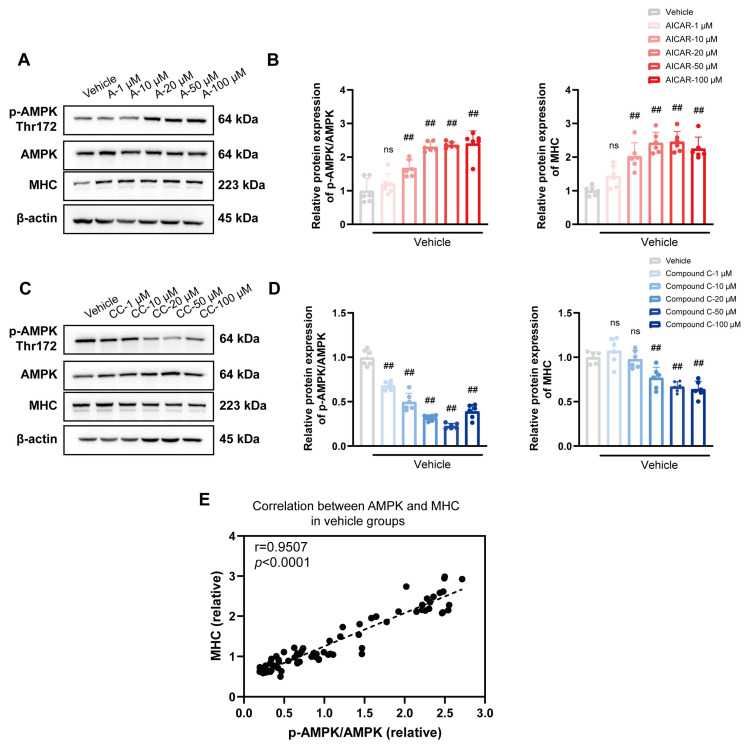
Relationship between p-AMPK/AMPK and MHC expression in vehicle groups after exposure to the (**A**,**B**) AMPK inhibitor AICAR and (**C**,**D**) AMPK agonist compound C. n = 6. ^##^ *p* < 0.01, compared with the vehicle group. ns, no significance. (**E**) Pearson correlation analysis with all dates in vehicle groups with r = 0.9507 and *p* < 0.0001.

**Figure 7:**
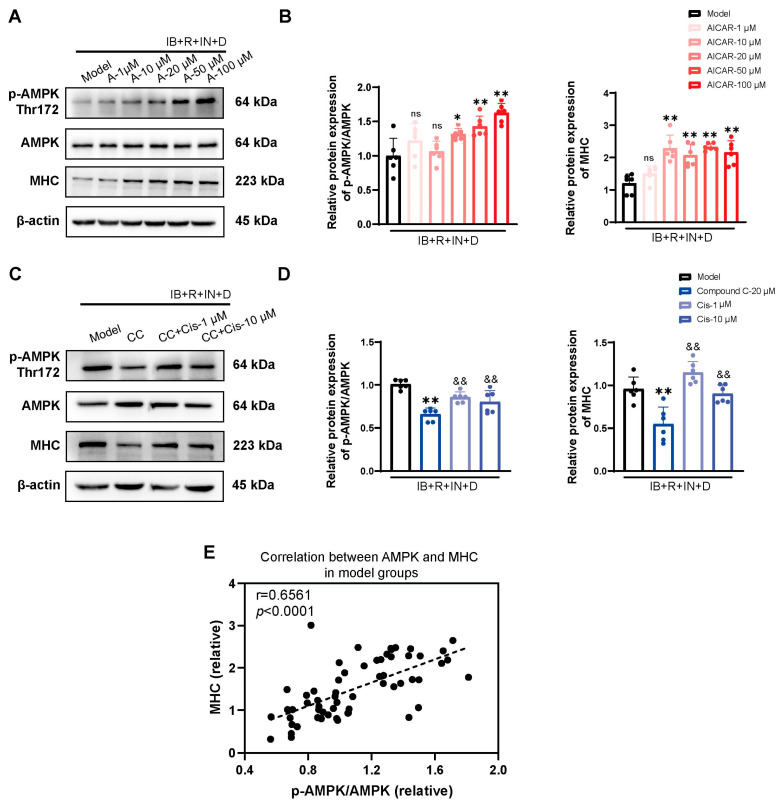
Relationship between p-AMPK/AMPK and MHC expression in model groups after exposure to the (**A**,**B**) AMPK inhibitor AICAR and (**C**,**D**) AMPK agonist compound C. n = 6. * *p* < 0.05, ** *p* < 0.01, compared with the model group; ^&&^ *p* < 0.01, compared with the compound C group. ns, no significance. (**E**) Pearson correlation analysis with all dates in model groups with r = 0.6561 and *p* < 0.0001.

To explore the relationship between MHC and AMPK further, the vehicle and model groups were exposed to the AMPK inhibitor compound C and the AMPK activator AICAR. Notably, we observed a positive correlation between MHC and AMPK in the vehicle and model groups (Figure 6E and Figure 7E). AICAR led to a concentration-dependent increase in the expression of both p-AMPK/AMPK and MHC (Figure 6 and Figure 7A,B). In contrast, in the presence of compound C, both p-AMPK/AMPK and MHC expression decreased (Figure 6C,D), and Cis effectively upregulated p-AMPK/AMPK and MHC expression (Figure 7C,D). Thus, the muscle-promoting effects of Cis may be mediated by the AMPK pathway.

### 3.6. Cis Effectively Reduces Intracellular Inflammation and Counteracts LPS-Induced Atrophic Effects

**Figure 8:**
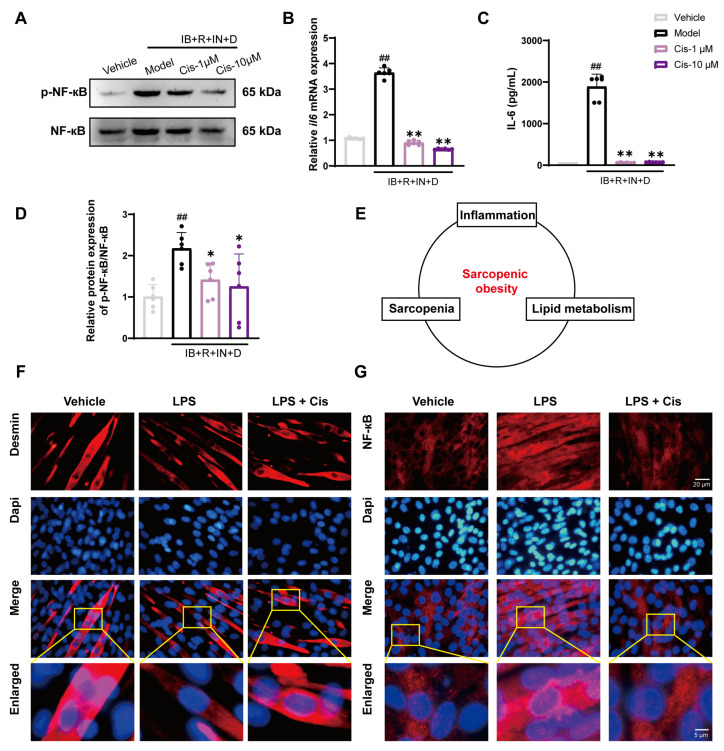
Expression of inflammation- and muscle-related markers after Cis addition in IB+R+IN+D and LPS models. (**A**,**D**) p-NF-κB/NF-κB expression in the IB+R+IN+D model. (**B**,**C**) *Il6* mRNA and Elisa expression in the IB+R+IN+D model. (**E**) The relationship between sarcopenia, inflammation, and lipid metabolism. (**F**,**G**) Immunofluorescence localization of the inflammatory marker NF-κB (Red) and muscle quantity marker Desmin (Red) in the LPS model. Nuclei were stained with DAPI (Blue). n = 6. ^##^ *p* < 0.01, compared with the vehicle group; * *p* < 0.05, ** *p* < 0.01, compared with the model group. ns, no significance.

To assess the relationship between abnormal lipid deposition and inflammation onset and the potential of molecular compounds to mitigate reactive inflammation, we assessed abnormal reactive inflammatory factor expression in our IB+R+IN+D and LPS models. The results demonstrated that Cis effectively reduced the level of abnormal reactive inflammatory factors produced (Figure 8B,C) and downregulated p-NF-κB/NF-κB protein expression (Figure 8A,D). These results suggested that Cis effectively attenuates the inflammatory response triggered by lipogenic differentiation induced by IB+R+IN+D. Next, we induced a novel LPS-induced cellular inflammation model; the results demonstrated that Cis alleviated muscle loss and counteracted inflammation, thereby reinforcing the pharmacological efficacy of Cis in skeletal muscles. This further highlighted the correlation among inflammation, sarcopenia, and lipid metabolism (Figure 8E). Immunofluorescence staining was used to localize the muscle quantity marker Desmin and the inflammation marker NF-κB (Figure 8F,G). Taken together, these results indicated that Cis effectively reduces inflammation, suggesting it could be a focus for further research.

## 4. Discussion

In this study, we identified a novel bioactive monomer, Cis, from natural plant sources. This compound demonstrated both safety and efficacy in reducing adipocyte lipid accumulation, and it enhanced skeletal muscle growth and differentiation processes. The findings suggested that Cis has considerable potential for SO prevention and treatment. Our preliminary investigation on the molecular mechanisms of action of Cis may provide valuable insights for the future development of therapeutics for metabolic and muscular disorders.

The potential correlation between muscle development and obesity risk is a compelling area of research. Skeletal muscle is a highly plastic tissue exhibiting remarkable responsiveness to various physiological signals. Notably, emerging evidence suggests that muscle composition may undergo developmental “programming” during critical periods, potentially predisposing individuals to obesity in their later life stages. The pathophysiological progression of SO is characterized by a complex interplay of various factors, including age-related muscle atrophy, concomitant adipose tissue accumulation, growth microenvironmental factor alterations, and chronic inflammatory stress responses. Notably, intramuscular lipid overload exerts detrimental effects on insulin pathways, muscle mass maintenance, and regenerative capacity. In contrast, maintaining optimal skeletal muscle mass mitigates obesity-related complications. Consequently, therapeutic interventions targeting both myogenesis enhancement and lipid metabolism regulation are crucial for promoting muscular hypertrophy and reducing adiposity. In other words, the most effective SO management strategy should concurrently address adipose tissue reduction and muscle mass augmentation via a dual-targeted approach.

Regarding fatty acid metabolism, the observed metabolic alterations are characterized by enhanced fatty acid uptake and storage coupled with impaired complete lipolytic processes, indicating dysregulated lipid turnover. Obesity-induced metabolic perturbation in skeletal muscle tissue leads to metabolic inflexibility, ectopic lipid deposition, and deleterious lipid intermediate generation. Excessive saturated fatty acid uptake exacerbates intramuscular lipid infiltration and frequently inhibits myogenesis [42]. In the present study, two classical adipogenic differentiation-induced cell models were established to evaluate the effects of Cis. Our initial analysis focused on the cytoprotective properties of Cis in myotube cells. The results revealed that Cis exhibits no significant cytotoxicity toward C2C12 cells but has proliferative-enhancing effects on them. Furthermore, Cis treatment resulted in a time-dependent increase in myotube diameter; moreover, it upregulated the expression of MHC, a specific myotube differentiation marker, particularly during the terminal phase of adipogenic differentiation.

We also observed a considerable lipid-lowering effect and amelioration of ectopic lipid deposition in skeletal muscle tissue. The pathological hallmark of adipogenic differentiation in C2C12 cells manifests as abnormal lipid accumulation and lipid droplet coalescence, characterized by increases in intracellular lipid droplet contents, along with elevated TCHO, TG, LDL, and HDL levels and upregulated PPARγ expression. Lipid mobilization represents a crucial mechanism underlying lipid catabolism and adipose tissue accumulation inhibition. Triglyceride metabolism regulation is mediated by key enzymes localized on lipid droplet surfaces, particularly ATGL, which catalyzes the initial step of triglyceride hydrolysis, as well as CPT1—a rate-limiting enzyme in fatty acid oxidation (FAO). Moreover, UCP1, a mitochondrial transporter predominantly expressed in brown adipose tissue, is pivotal in thermogenesis. Notably, studies reported that cafeteria diet-fed dams’ offspring exhibited reduced muscle cross-sectional area and fiber number, concomitant with increased PPARγ transcript levels in muscle tissue, indicating that alterations in PPARγ expression are associated with considerable muscular changes. Our experimental results indicated that Cis modulates lipid catabolic pathways and enhances lipid mobilization, possibly mechanistically contributing to its myotube proliferative effects. Thus, the lipid-modulating properties of Cis may be intrinsically linked to its myogenic potential through complex metabolic regulatory mechanisms.

Mitochondria are the key organelles that regulate lipid metabolism in skeletal muscle, and the main signs of mitochondrial dysfunction include membrane potential loss (ΔΨ) and increased ROS release [43,44]. Peroxisome proliferator-activated receptor gamma coactivator-1α (PGC-1α) is an important regulatory factor in mitochondrial biogenesis and plays a key role in increasing mitochondrial quality and activity [45]. As the core of energy metabolism, AMPK is closely related to the regulation of mitochondrial biogenesis and lipid metabolism [46]. In skeletal muscle, the AMPK/PGC-1α pathway has been verified to improve abnormal lipid metabolism by regulating mitochondrial function [47]. The AMPK/ACC1 pathway is a fundamental regulatory axis in lipid metabolism and thus a primary target in lipid-lowering therapeutic development [48]. Because pharmacological targets typically exhibit pronounced structural specificity, precise structural characterization of bioactive compounds is essential for elucidating their therapeutic mechanisms. Our molecular docking analyses revealed that Cis significantly enhances AMPK activity, as evidenced by the increased Thr172 phosphorylation of AMPKα, a well-established AMPK activation marker. Experimental data indicated that Cis effectively reduces ROS, enhances MMP, and upregulates the expression of PGC-1α for improving mitochondrial function. Furthermore, Cis induces ACC1 phosphorylation via AMPK activation, thereby inhibiting lipid synthesis. These findings provide mechanistic insights into the lipid-modulating properties of Cis, involving its interaction with the AMPK/ACC1 pathway.

To investigate the potential role of AMPK activation in myogenesis, we conducted systematic experiments by using the AMPK activator AICAR and the AMPK inhibitor compound C at varying concentrations. We found that AMPK activation has a significant regulatory role in myogenic differentiation. In particular, AMPK activation was noted to upregulate MHC expression, even under conditions of PPARγ activation in the presence of insulin. Furthermore, we observed that Cis effectively enhances MHC expression through AMPK phosphorylation, counteracting the inhibitory effects of compound C. These experimental results are consistent with those reported previously, confirming that the dual beneficial effects of Cis on lipid metabolism modulation and skeletal muscle development are mediated through AMPK signaling factor regulation.

Another notable observation in the current study was regarding the total protein expression profile of ACC, which prompted us to hypothesize that the observed phenomenon is associated with ACC’s distinct isoforms. Mammalian cells express two ACC isoforms with distinct functional roles: ACC1 primarily promotes fatty acid synthesis [49], whereas ACC2 inhibits lipid lipolysis [50]. Through quantitative analysis of ACC isoform expression in our IB+R+IN+D model, we noted significantly phosphorylated ACC1, which was effectively attenuated by Cis treatment. No significant alterations in ACC2 expression were observed across the experimental groups. Therefore, we have also shown great interest in whether Cis could affect the post-translational modification of ACC2, but based on the current experimental limitation, we think this is a good point to study in future studies. These findings corroborate previous studies demonstrating the predominant localization of ACC1 in adipocyte-rich tissues, where it plays a pivotal role in the de novo lipogenesis (DNL) pathways [51]. These observations provided preliminary evidence that the ameliorative effects of Cis on abnormal skeletal muscle lipid deposition are mediated, at least in part, via lipogenesis suppression. However, whether ACC1 is the primary molecular target of Cis warrants further investigation to fully elucidate the compound’s mechanism of action.

Previous studies have shown that AMPK is involved in the myogenic differentiation of C2C12 cells, but the results remain controversial. Ting Zhang et al. [52] reported that AMPK could regulate myogenesis through phosphorylating TET2 and effectively resist cell apoptosis reported by Carola et al. [53]. In contrast, Chia et al. [54] denied the positive regulatory role of AMPK in myogenic differentiation. In this study, Cis significantly enhanced *Mhc* transcription and protein expression in the model groups, indicating that Cis has a notable muscle-building effect. Furthermore, AMPK was strongly correlated with MHC, suggesting it may be a key target for SO management.

Our results revealed that elevated free fatty acids significantly activate NF-κB signaling and facilitate its nuclear translocation, exacerbating insulin resistance in skeletal muscle cells. This finding is particularly relevant given that SO patients exhibit elevated IL6 levels compared with healthy individuals [55], suggesting a crucial role of chronic inflammation in sarcopenia pathogenesis. The underlying mechanisms may involve multiple pathways, including enhanced skeletal muscle protein degradation, upregulation of atrogin 1 expression, suppression of insulin-like growth factor synthesis, and induction of skeletal muscle insulin resistance [56,57]. Studies have established a strong correlation between high-fat diet-induced obesity, insulin resistance, and NF-κB pathway activation [58]. Furthermore, Knudsen et al. [59] demonstrated that skeletal muscle-derived IL6 can attenuate AMPK phosphorylation, whereas AMPKα phosphorylation at Thr172 exerts anti-inflammatory effects. This is supported by evidence showing that lipopolysaccharide-induced AMPK dephosphorylation increases inflammatory responses and the upregulation of inflammatory mediators [60]. Emerging evidence suggests that proinflammatory signaling cascades are pivotal in modulating skeletal muscle energy homeostasis. In obesity, both proinflammatory and stress-activated signaling pathways, the critical mediators of obesity-related metabolic dysfunction and insulin resistance pathogeneses, are upregulated. At the molecular level, activation of these pathways in skeletal muscle tissue triggers a cascade of cellular events that lead to increased apoptosis and muscular atrophy, eventually contributing to the deterioration of muscle mass and function [61]. The current results demonstrated that the p-NF-κB/NF-κB ratio, as well as *IL6* mRNA and protein expression, were significantly upregulated in the model groups after quadruple-induced differentiation, all of which could be reduced Cis, indicating its anti-inflammatory effects.

In summary, Cis ameliorates pathological lipid accumulation, enhances myogenic differentiation, and prevents SO by modulating the skeletal muscle AMPK pathway, which simultaneously regulates ACC-mediated lipid metabolism and MHC-dependent myogenesis. Furthermore, because Cis exhibited significant anti-inflammatory properties in obesity models, it is a potential multitarget therapeutic agent. While our finding provides valuable insights into the therapeutic potential of Cis, its side effects and pharmacokinetic characteristics remain to be fully elucidated. Future investigations will focus on systematically characterizing the compounds’ in vivo pharmacological activity, particularly their absorption–distribution patterns and metabolic transformation pathways, which constitute essential prerequisites for clinical translation.

## 5. Conclusions

Our experimental findings indicated that Cis exerts major therapeutic effects through AMPK pathway modulation, ameliorating pathological lipid accumulation in C2C12 cells. Thus, Cis is a promising pharmacological candidate for SO treatment. Furthermore, we identified a crucial positive regulatory mechanism between AMPK activation and MHC expression; this mechanism may be a therapeutic target with broader applications in metabolic regulation, including adipose tissue reduction and muscle mass augmentation.

Our findings provide a strong rationale for further investigation into the comprehensive pharmacological applications of Cis in lipid metabolism and muscular disorders. Moreover, the mechanistic insights establish a robust scientific foundation for developing other innovative therapeutic strategies targeting the complex interplay between metabolic dysregulation and muscular degeneration in various pathological conditions, in addition to SO.

## Figures and Tables

**Table 1:** PCR primer sequences used here.

Gene	Forward Primer (5′–3′)	Reverse Primer (3′–5′)	Length (bp)
*Mhc*	CAGACGGAGAGGAGCAGGAAG	CTTGGTGTTGATGAGGCTGGTG	102
*Il6*	CTCCCAACAGACCTGTCTATAC	CCATTGCACAACTCTTTTCTCA	97
*Actb*	CTACCTCATGAAGATCCTGACC	CACAGCTTCTCTTTGATGTCAC	90

## Data Availability

Data will be made available from the corresponding author upon reasonable request.

## Abbreviations

SO	Sarcopenic Obesity
Cis	Cistanoside F
PPARγ	Peroxisome proliferator-activated receptor gamma
ATGL	Adipose triglyceride lipase
CPT1	Carnitine palmitoyltransferase 1
UCP1	Uncoupling protein 1
IL	Interleukin
NF-κB	Nuclear factor-kappa B
AMPK	AMP-activated protein kinase
ACC	acetyl-CoA carboxylase
MHC	Myosin heavy chain
IB+R+IN+D	Isobutylmethylxanthine, dexamethasone, insulin, rosiglitazone (R)
PA	Palmitic acid
TG	Triglyceride
TCHO	Total Cholesterol
LDL	Low Density Lipoprotein
HDL	High Density Lipoprotein
PGC-1α	Peroxisome proliferator-activated receptor gamma coactivator-1α
